# Is Obesity Associated With an Increased Risk of Complications After Surgical Management of Acetabulum and Pelvis Fractures? A Systematic Review

**DOI:** 10.5435/JAAOSGlobal-D-21-00058

**Published:** 2021-04-19

**Authors:** Peter N. Mittwede, Christopher M. Gibbs, Jaimo Ahn, Patrick F. Bergin, Ivan S. Tarkin

**Affiliations:** From the Department of Orthopaedic Surgery, University of Pittsburgh, Pittsburgh, PA (Dr. Mittwede, Dr. Gibbs, and Dr. Tarkin); the Department of Orthopaedic Surgery, University of Michigan, Ann Arbor, MI (Dr. Ahn); and the Department of Orthopaedic Surgery, University of Mississippi Medical Center, Jackson, MS (Dr. Bergin).

## Abstract

**Background:**

When considering surgical fixation of acetabulum and pelvis fractures in patients with obesity, a thorough understanding of the risks of potential complications is important. We performed a systematic review to evaluate whether obesity is associated with an increased risk of complications after surgical management of acetabulum and pelvis fractures.

**Methods:**

We searched PubMed/MEDLINE, EMBASE, and the Cochrane Library for studies published through December 2020 that reported the effect of increased body mass index (BMI) or obesity on the risk of complications after surgical treatment of acetabulum and pelvis fractures.

**Results:**

Fifteen studies were included. Eight of the 11 studies that included infection or wound complication as end points found that increased BMI or some degree of obesity was a significant risk factor for these complications. Two studies found that obesity was significantly associated with loss of reduction. Other complications that were assessed in a few studies each included venous thromboembolism, nerve palsy, heterotopic ossification, general systemic complications, and revision surgery, but obesity was not clearly associated with those outcomes.

**Conclusions:**

Obesity (or elevated BMI) was associated with an increased risk of complications—infection being the most commonly reported—after surgical management of acetabulum and pelvis fractures, which suggests the need for increased perioperative vigilance.

Obesity is a major public health concern in the United States, and its prevalence is increasing.^1^ Its contribution to various chronic diseases within the context of metabolic syndrome, which includes hypertension, insulin resistance, and hyperlipidemia, is well known.^2^ In the general trauma literature, obesity has been associated with increased morbidity and mortality rates.^3,4^ The impact of obesity on orthopaedic surgery has also been increasingly realized,^5,6^ and although preoperative patient optimization and weight loss have been areas of focus with elective procedures,^7^ this is not possible with fracture care because of the acute need for surgical intervention.

The technical and logistical challenges posed by obesity are considerable, particularly with the surgical treatment of acetabulum and pelvis fractures.^8,9^ High-energy acetabulum and pelvis fractures are complex injuries most commonly treated by fellowship-trained orthopaedic trauma surgeons, and appropriate management of these patients is critical to optimize outcomes and minimize complications. Although patients with obesity with acetabulum and pelvis fractures would benefit from an injury-specific treatment paradigm, such guidelines have not been established. Until then, a clear understanding of the relationship between obesity and complications after surgery will facilitate preoperative and perioperative planning and help guide future studies.

Thus, we performed a systematic review to answer the following questions: (1) Is obesity associated with an increased risk of complications after surgical management of acetabulum and pelvis fractures? (2) What complications are patients with obesity at an increased risk for after surgery for acetabulum and pelvis fractures?

## Methods

### Literature Search

**Figure 1:**
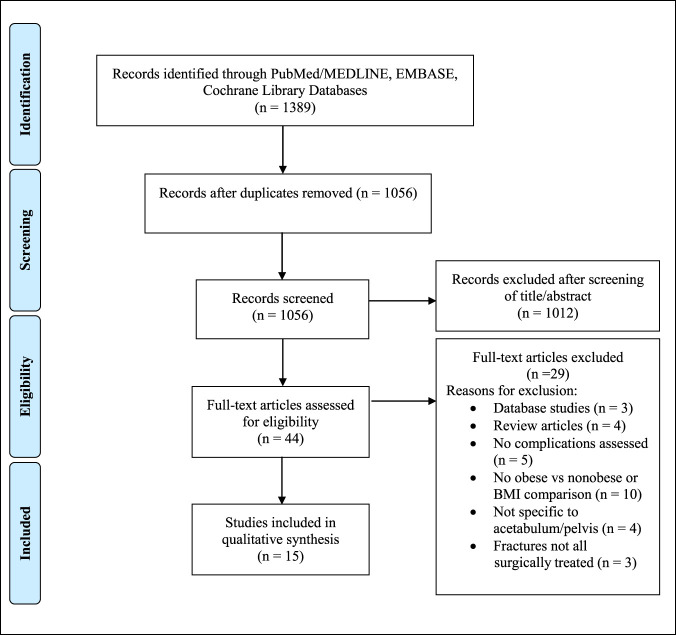
Preferred Reporting Items for Systematic Reviews and Meta-Analyses (PRISMA) flow diagram highlighting the search strategy and selection method for studies included in this systematic review.

The literature review was performed according to the Preferred Reporting Items for Systematic Reviews and Meta-Analyses (PRISMA) guidelines^10^ with the use of a PRISMA checklist. Following the establishment of our protocol, the systematic review was registered in the PROSPERO database (CRD178645) (crd.york.ac.uk/PROSPERO). We used the following medical subject headings (MeSH) and key terms to search in article titles, abstracts, and keywords: (obesity OR obese OR body mass index) AND (pelvis OR pelvic ring OR acetabulum OR acetabular) AND (complication OR mortality OR infection OR failure OR reoperation OR thromboembolism OR nonunion). We performed the search in the PubMed/MedLine, EMBASE, and Cochrane Library databases (last accessed December 15, 2020) to identify studies for potential inclusion and manually searched the reference lists of the included studies and related review articles to identify additional articles for consideration of inclusion. Our overall study selection flow diagram is shown in Figure 1.

### Inclusion and Exclusion Criteria

Two authors independently performed the literature review to include articles published from the inception of the respective databases through December 15, 2020, and the decision to include/exclude articles was made by consensus. Articles met the inclusion criteria if they (1) evaluated complications after surgical management of acetabulum or pelvis fractures and (2) compared the risk of complications based on the level of obesity or body mass index (BMI). Exclusion criteria were review articles, database studies, studies published in a language other than English, studies that described complication rates in a general orthopaedic trauma population that was not specific to acetabulum or pelvis trauma, and studies that included acetabulum or fractures treated nonsurgically.

### Data Extraction

**Figure 2:**
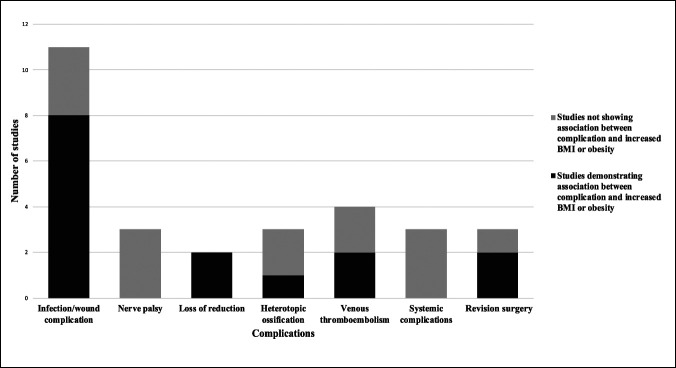
Graph highlighting the number of studies that have evaluated the association between an increased body mass index (BMI) or obesity and complications after acetabulum or pelvis surgery. Black indicates the number of studies demonstrating an association between complication and increased BMI or obesity, whereas gray indicates the number of studies not showing an association between complication and increased BMI or obesity. Venous thromboembolism includes either deep vein thrombosis or pulmonary embolism and systemic complications include respiratory complications, renal insufficiency, cardiac arrhythmias, and vascular complications.

Data were extracted from the articles and placed in a secure file. The data relevant to BMI or degree of obesity being associated with a particular complication were collected. All complications that the authors presented were recorded, and these included infection/wound complication (these were combined for ease of presentation and because they were variably defined between studies), nerve palsy, loss of reduction, heterotopic ossification (HO), deep vein thrombosis, pulmonary embolism, other systemic complications, and revision surgery. For the purposes of Figure 2, venous thromboembolism included either deep vein thrombosis or pulmonary embolism, and systemic complications included respiratory complications, renal insufficiency, cardiac arrhythmias, and vascular complications. We also collected rates of overall complications when documented.

### Assessment of Study Quality

Two authors independently reviewed the included articles to assess their quality (Table 1). The methodological index for nonrandomized studies (MINORS) instrument was used to grade the methodological quality of the studies.^11^ Briefly, the MINORS score assigns points based on responses to 12 items. Each item was given a score of 2 (reported and adequate), 1 (reported but inadequate), or 0 (not reported), with comparative studies achieving a maximum of 24 points and noncomparative studies achieving a maximum of 16 points (4 of 12 of the items are only relevant to comparative studies). Higher scores with the MINORS instrument are associated with higher-quality studies with a lower risk of bias.

**Table 1:** Characteristics of the Included Studies

Authors	Year	Journal	Study Design	No. of Patients	Fractures Included	Average MINORS Score^a^
Ding et al^20^	2018	*Journal of Orthopaedic Trauma*	Retrospective cohort	791	Acetabulum	15/24
Hupel et al^24^	1998	*Journal of Trauma*	Retrospective case-control	42	Pelvis	15/24
Iqbal et al^12^	2017	*Hip & Pelvis*	Retrospective case-control	261	Acetabulum	17/24
Jaeblon et al^13^	2018	*Journal of Orthopaedic Trauma*	Retrospective case-control	161	Pelvis and acetabulum	16/24
Karunakar et al^14^	2005	*Journal of Bone and Joint Surgery*	Retrospective cohort	169	Acetabulum	16/24
Li et al^15^	2015	*Surgical Infections*	Retrospective case-control	338	Acetabulum	13/24
Mears et al^23^	2003	*Clinical Orthopaedics and Related Research*	Retrospective cohort	424	Acetabulum	8/16
Mourad et al^25^	2012	*International Journal of Radiation Oncology, Biology, Physics*	Retrospective case-control	395	Acetabulum	16/24
Porter et al^16^	2008	*Journal of Orthopaedic Trauma*	Retrospective cohort	435	Acetabulum	15/24
Porter et al^26^	2008	*Obesity Surgery*	Retrospective cohort	288	Pelvis	15/24
Sagi et al^17^	2013	*Journal of Orthopaedic Trauma*	Retrospective case-control	97	Pelvis and acetabulum	14/24
Sems et al^18^	2010	*Journal of Orthopaedic Trauma*	Retrospective cohort	182	Pelvis	17/24
Shaath et al^22^	2020	*Injury*	Retrospective cohort	333	Acetabulum	15/24
Suzuki et al^19^	2010	*Injury*	Retrospective case-control	326	Acetabulum	12/24
Vincent et al^21^	2014	*Journal of Orthopaedics*	Retrospective cohort	81	Acetabulum	11/24

a See Supplemental Digital Content for detailed break-down of MINORS (methodological index for nonrandomized studies) scores for each study

## Results

### Search Results

Our database searches initially yielded 1,389 results (Figure 1). After removing duplicates, authors PM and CG screened the titles and/or abstracts of 1,066 articles. This screen led to the removal of 1,012 articles, and we reviewed the full texts of the remaining 44. After further review, we excluded an additional 29 studies based on our exclusion criteria, and we were left with 15 for inclusion in the final analysis.

All 15 included studies were retrospective (Table 1). We found a wide variation in how patients' body weight was evaluated as a risk factor for complications after acetabulum and pelvis surgery, with some studies evaluating increased BMI on a continuous scale and others comparing patients with obesity or morbid obesity with patients who were nonobese or nonmorbidly obese (with a variable BMI cutoff for obesity and morbid obesity used between studies). Nonetheless, 13 of the 15 studies found that elevated BMI or some degree of obesity was associated with an increased risk of at least one type of complication (Table 2).

**Table 2:** Overview of the Findings of the Included Studies

Authors	Obesity/BMI Categorization Used	Complication Type Evaluated	Notable Findings
Ding et al^20^	BMI—continuous scale	Infection, revision surgery	Mean BMI in the infection group, 31.2 kg/m^2^; mean BMI in the no-infection group, 29 kg/m^2^ (*P* = 0.03)^a^ Increased BMI not a significant risk factor for infection on multivariate analyses: OR 1.03 (*P* = 0.06) BMI not significantly associated with the risk of revision surgery
Hupel et al^24^	Nonobese (BMI <85th percentile), obese (BMI >85th percentile)	Inability to obtain/maintain reduction	Increased rate of inability to obtain/maintain reduction in the obese group (5/10, 50%) versus nonobese group (2/32, 6.3%) (*P* < 0.005)^a^
Iqbal et al^12^	BMI—continuous scale	Infection	Mean BMI in the infection group, 32.4 kg/m^2^; mean BMI in the no-infection group, 27.9 kg/m^2^ (*P* = 0.004)^a^ Increased BMI a risk factor for infection on multivariate analyses: OR 14.2 (*P* = 0.003)^a^
Jaeblon et al^13^	Nonobese (BMI <30 kg/m^2^), obese (BMI ≥30 kg/m^2^), morbidly obese (BMI ≥40 kg/m^2^), waist-hip ratio	Wound complication	Mean BMI in the wound complication group, 36.3 kg/m^2^; mean BMI in the no-wound-complication group, 30 kg/m^2^ (*P* < 0.001)^a^ BMI ≥30 kg/m^2^ a risk factor for wound complication on multivariate analyses: OR 3.82 (*P* = 0.02)^a^ BMI ≥40 kg/m^2^ a risk factor for wound complication on multivariate analyses: OR 4.58 (*P* = 0.008)^a^ Waist-hip ratio a risk factor for wound complication on multivariate analyses: OR 23.41 (*P* = 0.003)^a^
Karunakar et al^14^	Normal (BMI <25 kg/m^2^), overweight (BMI ≥25 to <30 kg/m^2^), obese (BMI ≥30 to <40 kg/m^2^), and morbidly obese (BMI ≥40 kg/m^2^)	Infection, nerve palsy, HO, DVT, and PE	Increased BMI (continuous variable) a risk factor for infection (*P* = 0.002)^a^ and DVT (*P* = 0.03)^a^ BMI ≥30 kg/m^2^ a risk factor for DVT on multivariate analyses: OR 2.6 (*P* = 0.03)^a^ BMI ≥40 kg/m^2^ a risk factor for infection on multivariate analyses: OR 5 (*P* = 0.05)^a^ BMI ≥30 kg/m^2^ not significantly associated with the risk of nerve palsy, HO, or PE
Li et al^15^	BMI—continuous scale	Infection	Mean BMI in the infection group, 27.3 kg/m^2^; mean BMI in the no-infection group, 22.6 kg/m^2^ (*P* = 0.000)^a^ Increased BMI a risk factor for infection on multivariate analyses: OR 1.43 (*P* = 0.021)^a^
Mears et al^23^	Nonmorbidly obese (less than double the expected BMI), morbidly obese (more than double the expected BMI)	Infection, HO, clinical results	All 13 cases of grade III and IV HO and all 10 cases of deep infection occurred in patients with morbid obesity (statistical comparisons not performed) Morbid obesity a risk factor for fair or poor clinical results (*P* < 0.001)^a^
Mourad et al^25^	Underweight (BMI <18.5 kg/m^2^), normal (BMI 18.5-24.9 kg/m^2^), overweight (BMI 25-29.9 kg/m^2^), obese (BMI >30 kg/m^2^)	HO	Increased incidence of HO with increasing BMI: <18.5 kg/m^2^ (0%), 18.5-24.9 kg/m^2^ (6%), 25-29.9 kg/m^2^ (19%), >30 kg/m^2^ (31%) Increased BMI a risk factor for HO development (including grade III and IV HO) on multivariate analyses (*P* < 0.0001)^a^
Porter et al^16^	Nonmorbidly obese (BMI <40 kg/m^2^), morbidly obese (BMI ≥40 kg/m^2^)	Wound complication, overall complications	Increased wound complication rate with BMI ≥40 kg/m^2^ (46%) versus BMI <40 kg/m^2^ (12%) (*P* < 0.0001)^a^ Increased overall complication rate with BMI ≥40 kg/m^2^ (63%) versus BMI <40 kg/m^2^ (24%) (*P* < 0.0001)^a^
Porter et al^26^	Nonobese (BMI <30 kg/m^2^), obese (BMI >30 kg/m^2^)	Overall complications, revision surgery	Increased overall complication rate with BMI >30 kg/m^2^ (39%) versus BMI <30 kg/m^2^ (19%) (*P* < 0.0001)^a^ Increased rate of revision surgery with BMI >30 kg/m^2^ (31%) versus BMI <30 kg/m^2^ (16%) (*P* < 0.005)^a^
Sagi et al^17^	Nonobese (BMI <30 kg/m^2^), obese (BMI >30 kg/m^2^)	Infection	Increased infection rate with BMI >30 kg/m^2^ (33.3%) versus BMI <30 kg/m^2^ (6%) (*P* = 0.001)^a^ BMI >30 kg/m^2^ a risk factor for infection on univariate analyses: OR 7.83 (*P* < 0.05)^a^
Sems et al^18^	Nonobese (BMI <30 kg/m^2^), obese (BMI >30 kg/m^2^)	Infection, loss of reduction, revision surgery, DVT, PE, pneumonia, and nerve injury	Increased infection rate with BMI >30 kg/m^2^ (22.9%) versus BMI kg/m^2^ <30 kg/m^2^ (3.7%) (*P* = 0.0002)^a^ Increased rate of loss of reduction with BMI >30 kg/m^2^ (31.3%) versus BMI kg/m^2^ <30 (6%) (*P* < 0.0001)^a^ Increased rate of revision surgery with BMI >30 kg/m^2^ (33.3%) versus BMI <30 kg/m^2^ (9.7%) (*P* = 0.0003)^a^ Increased BMI not significantly associated with the risk of DVT, PE, pneumonia, or nerve injury
Shaath et al^22^	Normal (BMI <25 kg/m^2^), preobese (BMI 25-29.9 kg/m^2^), obese (BMI 30-39.9 kg/m^2^), morbidly obese (BMI ≥40 kg/m^2^)	Infection, DVT, PE, mortality, medical complication, nerve injury, and HO	BMI ≥40 (10%) infection rate not significantly greater than the infection rate in normal and preobese groups combined, BMI <29.9 kg/m^2^ (*P* = 0.08) BMI ≥40 kg/m^2^ a risk factor for PE compared with BMI <29.9 kg/m^2^ (*P* < 0.01) Increased BMI not significantly associated with the risk of DVT, mortality, medical complication, nerve injury, or HO
Suzuki et al^19^	BMI—continuous scale	Infection	Mean BMI in the infection group, 33 kg/m^2^; mean BMI in the no-infection group, 26.9 kg/m^2^ (*P* = 0.001)^a^ Increased BMI a risk factor for infection on multivariate analyses (per 1 kg/m^2^: OR 1.1 (*P* = 0.015)^a^
Vincent et al^21^	Nonmorbidly obese (BMI <35 kg/m^2^), morbidly obese (BMI ≥35 kg/m^2^)	Infection, DVT, PE, systemic complications, and sciatic nerve palsy	Infection rate with BMI ≥35 kg/m^2^ (20%) versus BMI <35 kg/m^2^ (8.3%) (*P* = 0.155) Increased BMI not significantly associated with the risk of DVT, PE, systemic complications, or sciatic nerve palsy

BMI = body mass index, DVT = deep vein thrombosis, HO = heterotopic ossification, OR = odds ratio, PE = pulmonary embolism

a Statistically significant difference observed.

### Complications

Eight of the 11 studies that included infection/wound complication as an end point found a significant association with increased BMI or some degree of obesity,^12,13,14,15,16,17,19^ whereas the other three studies also showed an association in the same direction, which was not statistically significant^20-22^ (Figure 2). One other study^23^ found that deep infections and advanced HO developed only in the morbidly obese group, and although the authors observed significantly worse clinical outcomes in patients with morbid obesity compared with patients with nonmorbid obesity, they did not statistically compare the rates of infection or HO between the groups. Two studies evaluated loss of reduction as an end point after pelvic ring fixation, and both found that obesity was significantly associated with this complication.^18,24^ Other complications that were assessed included venous thromboembolism,^14,18,21,22^ nerve palsy,^14,18,21^ HO,^14,22,25^ general systemic complications,^18,21,22^ and revision surgery,^18,20,26^ but conflicting results were found regarding the effect of obesity. The risks of overall complications based on the BMI or degree of obesity was assessed in multiple studies and are presented in Table 2 to highlight the findings (but not included in Figure 2 as they do not represent specific complications).

### Quality of the Included Studies

Because the MINORS scores ranged from 11 to 17 of a maximum of 24 for the included comparative studies (summary in Table 1, details in Table, Supplemental Digital Content, http://links.lww.com/JG9/A120), reflecting relatively low quality of evidence, high risk of bias, and heterogeneity between the studies, we did not perform a meta-analysis.

## Discussion

The goal of our systematic review was to assess whether obesity was associated with an increased risk of complications after surgical management of acetabulum and pelvis fractures, and if so, to identify the specific associated complications. Fifteen retrospective studies were evaluated as no prospective studies have been performed on this topic. We found that the methods for evaluating whether obesity or elevated BMI was associated with complications were highly variable between studies. Infection was the most commonly studied complication, with eight of the 11 studies showing that elevated BMI or some degree of obesity was associated with infection, with the other three showing a nonstatistically significant association to this effect. Two studies found that obesity was associated with loss of reduction after pelvic ring fixation. For other complications, including nerve palsy, venous thromboembolism, HO, systemic complications, and revision surgery, obesity was either not found to be associated, or the results between studies were inconsistent.

This study had several limitations, some of which highlight the shortcomings of the literature on this topic. First, although we chose to focus primarily on surgically treated acetabulum and pelvis fractures to answer specific questions, it is also important to consider the effects of obesity on fractures not treated with surgery. Several studies have evaluated the role of BMI on complication rates after acetabulum/pelvis trauma with the inclusion of patients treated both with and without surgery.^27-29^ Although these more inclusive studies are valuable, they may have included injuries that did not meet surgical indications, patients too sick to have surgery, or had other important confounding issues. This constraint is also a limitation of database studies, which is why we chose to exclude them from this analysis despite the advantages of their large size. It is worth noting that all three database studies on this topic did show an increased risk of various complications in patients with obesity or morbid obesity with acetabulum or pelvis trauma.^30-32^ A second limitation is that the definition of obesity and morbid obesity was not consistent between studies, which makes it difficult to conclude what degree of obesity or elevated BMI actually contributes to the increased risk of complications. We recommend that the standard World Health Organization classification of obesity be used in all studies reporting BMI values^33^ and comparisons be made based on these subgroups. It is also important to consider that obesity is highly heterogeneous,^34^ and it is likely that the location of the adipose tissue can affect complication rates and outcomes. A patient who is obese based on the BMI scale because of being extremely muscular may have a different risk profile than one with the same BMI due to excessive fatty tissue. Thus, other variables, such as percentage body fat, thickness of the fat layer at the incision site, or the waist-hip ratio^13^ should be evaluated to assess the true effect of various degrees of obesity. Third, we combined both acetabulum and pelvis fractures treated with surgery because these injuries have been frequently reported together in the literature likely because most studies to date have used data from a single or a small number of institutions, and combining them helped increase the power of the studies. It should be recognized, however, that these are disparate injuries, and tremendous heterogeneity can exist even within the various subtypes of acetabulum and pelvis fractures. To answer precise questions and alter treatment approaches, larger studies evaluating specific injury types would be helpful, and multicenter studies will likely be needed to achieve the goal. Fourth, the included studies did not differentiate between wound complications and infections, so these were combined for the purposes of this review. However, future studies should use clear and consistent definitions and attempt to differentiate between superficial and deep infections. Finally, another limitation of this review was that all of the studies were retrospective. Thus, they were at a high risk for various types of bias and were of a lower level of evidence. Larger, higher-quality studies that are prospective will help to answer not only more specific questions about the role of obesity in contributing to the risk of complications but also how to appropriately manage patients with obesity to reduce complication rates.

Despite these limitations, the existing literature does show overall (and fairly consistently so) that obesity or elevated BMI is associated with various complications after surgical management of acetabulum and pelvis fractures. Infection has been the most frequently studied complication, with only a few studies each comparing the risk of loss of reduction, nerve palsy, HO, venous thromboembolism, systemic complications, and revision surgery. Although the data on infection are relatively convincing within the context of the retrospective studies available, additional studies are needed to elucidate the role that obesity may play in contributing to these other complications. The most recent study on this topic^22^ showed that complication rates (including infection) can be minimized with adherence to strict protocols and excellent surgical technique, which is encouraging.

The data in these studies highlight important unanswered questions. Various studies have evaluated complication rates after specific surgical approaches to the acetabulum and pelvis,^35-37^ but the optimal approaches and treatment methods in patients with obesity have not been determined. Goals of care must be established on individualized bases to maximize functionality while avoiding complications. Although the conventional treatment paradigm for patients with unstable acetabulum and pelvis fractures is anatomic reduction and internal fixation, this option may not be advisable in all patients if the risk profile is too high. Consensus should be sought on what fracture patterns or types absolutely require surgical intervention in patients with obesity, particularly in those who are morbidly obese. We believe that major goals of treatment should be to obtain a concentrically reduced, stable hip and a grossly stable pelvic ring to minimize systemic complications and optimize long-term musculoskeletal function. Within this context, a variety of treatment options should be considered, including closed management, percutaneous approaches, open reduction and internal fixation, and delayed arthroplasty. When open reconstruction is deemed prudent, the choice of surgical exposure should be chosen thoughtfully.

Perioperative approaches to minimize risk of complications in patients with obesity should be studied, ideally with prospective studies. It has been shown that despite having increased weight and adipose tissue, patients with obesity are frequently malnourished,^38^ which can affect healing potential, so it may be that nutritional optimization in the perioperative period could help reduce the risk of wound complications. One study showed that among 19 patients with morbid obesity who underwent acetabulum surgery and had an incisional vacuum placed, none developed an infection.^39^ The effectiveness of this method should be more rigorously evaluated in patients with obesity in the future, along with techniques such as drains and specific soft-tissue handling and closure methods. Given that multiple studies have observed that patients with obesity may be at an increased risk of loss of reduction and revision surgery after pelvic ring fixation, postoperative weight-bearing and rehabilitation protocols should be tailored to the individual patient and may need to be altered in individuals with obesity. If initial conservative management and delayed arthroplasty are pursued, optimizing the patient through prehabilitation (and potentially weight loss) could potentially improve outcomes. The role and efficacy of local antibiotics should be evaluated, with attention paid to dosing based on the degree of obesity.

In conclusion, in our systematic review of the literature, we found that the existing retrospective studies on this topic show that obesity or increased BMI is associated with various complications, particularly infection, after surgical fixation of acetabulum and pelvis fractures. This trend suggests that patients with obesity with these injuries may require a unique treatment paradigm. However, this study also highlights the low level of evidence of the existing literature and the large amount of heterogeneity between the studies. Additional studies are needed to help guide orthopaedic trauma surgeons in the optimal management of complex acetabulum and pelvis fractures in patients with obesity.

## Supplementary Material

SUPPLEMENTARY MATERIAL
