# Supplementary material for: Is Obesity Associated With an Increased Risk of Complications After Surgical Management of Acetabulum and Pelvis Fractures? A Systematic Review
Source: J Am Acad Orthop Surg Glob Res Rev. 2021 Apr 19;5(4):e21.00058. doi: 10.5435/JAAOSGlobal-D-21-00058 (PMC8057757; doi:10.5435/JAAOSGlobal-D-21-00058)
Supplement: SUPPLEMENTARY MATERIAL [file jagrr-5-e21.00058-s001.docx]

| **Supplemental Digital Content.** Methodological index for nonrandomized studies (MINORS) scores of the included studies | | | | | | | | | | | | | |
| --- | --- | --- | --- | --- | --- | --- | --- | --- | --- | --- | --- | --- | --- |
| **Study** | **Clearly stated aim** | **Inclusion of consecutive patients** | **Prospective collection of data** | **Endpoints appropriate to aim of study** | **Unbiased assessment of study endpoint** | **Follow-up period appropriate to aim of study** | **Loss to follow-up of less than 5%** | **Prospective calculation of sample size** | **Adequate control group** | **Contemporary groups** | **Baseline equivalence of groups** | **Adequate statistical analyses** | **Total** |
| Ding et al^20^ | 2 | 2 | 0 | 2 | 0 | 1 | 1 | 0 | 2 | 2 | 1 | 2 | 15/24 |
| Hupel et al^24^ | 2 | 0 | 1 | 2 | 0 | 2 | 1 | 0 | 2 | 2 | 1 | 2 | 15/24 |
| Iqbal et al^12^ | 2 | 2 | 0 | 2 | 0 | 2 | 2 | 0 | 2 | 2 | 1 | 2 | 17/24 |
| Jaeblon et al^13^ | 2 | 2 | 0 | 2 | 1 | 1 | 0 | 0 | 2 | 2 | 2 | 2 | 16/24 |
| Karunakar et al^14^ | 2 | 2 | 0 | 2 | 0 | 2 | 1 | 0 | 2 | 2 | 1 | 2 | 16/24 |
| Li et al^15^ | 2 | 2 | 0 | 2 | 0 | 0 | 0 | 0 | 2 | 2 | 1 | 2 | 13/24 |
| Mears et al^23^ | 1 | 2 | 0 | 2 | 0 | 2 | 1 | 0 | N/A | N/A | N/A | N/A | 8/16 |
| Mourad et al^25^ | 2 | 2 | 0 | 2 | 0 | 2 | 1 | 0 | 2 | 2 | 1 | 2 | 16/24 |
| Porter et al^16^ | 2 | 2 | 1 | 2 | 0 | 2 | 0 | 0 | 1 | 2 | 1 | 2 | 15/24 |
| Porter et al^26^ | 2 | 2 | 1 | 2 | 0 | 2 | 0 | 0 | 1 | 2 | 1 | 2 | 15/24 |
| Sagi et al^17^ | 2 | 1 | 0 | 2 | 0 | 1 | 0 | 0 | 2 | 2 | 2 | 2 | 14/24 |
| Sems et al^18^ | 2 | 2 | 0 | 2 | 0 | 2 | 2 | 0 | 2 | 2 | 1 | 2 | 17/24 |
| Shaath et al^22^ | 2 | 2 | 0 | 2 | 2 | 0 | 0 | 0 | 2 | 2 | 1 | 2 | 15/24 |
| Suzuki et al^19^ | 2 | 1 | 0 | 2 | 0 | 0 | 0 | 0 | 2 | 2 | 1 | 2 | 12/24 |
| Vincent et al^21^ | 2 | 1 | 0 | 1 | 0 | 0 | 0 | 0 | 2 | 2 | 1 | 2 | 11/24 |

Subsection scores: 2 = reported and adequate, 1 = reported but inadequate, 0 = not reported
